# Archaea Are Rare and Uncommon Members of the Mammalian Skin Microbiome

**DOI:** 10.1128/mSystems.00642-21

**Published:** 2021-07-20

**Authors:** Alexander K. Umbach, Ashley A. Stegelmeier, Josh D. Neufeld

**Affiliations:** a Department of Biology, University of Waterloo, Waterloo, Ontario, Canada; b Department of Pathobiology, University of Guelph, Guelph, Ontario, Canada; State University of Maringá

**Keywords:** archaea, mammalian skin, rare biosphere, built environment, microbiome, 16S RNA, gene sequencing

## Abstract

Although previous research demonstrates that skin-associated archaea are rarely detected within human skin microbiome data, exist at relatively low abundance, and are primarily affiliated with the *Methanobacteriota* and *Halobacteriota* phyla, other studies suggest that archaea are consistently detected and relatively abundant on human skin, with skin “archaeomes” dominated by putative ammonia oxidizers of the *Nitrososphaeria* class (*Thermoproteota* phylum, formerly *Thaumarchaeota*). Here, we evaluated new and existing 16S rRNA gene sequence data sourced from mammalian skin and skin-associated surfaces and generated with two commonly used universal prokaryotic primer sets to assess archaeal prevalence, relative abundance, and taxonomic distribution. Archaeal 16S rRNA gene sequences were detected in only 17.5% of 1,688 samples by high-throughput sequence data, with most of the archaeon-positive samples associated with nonhuman mammalian skin. Only 5.9% of human-associated skin sample data sets contained sequences affiliated with archaeal 16S rRNA genes. When detected, the relative abundance of sequences affiliated with archaeal amplicon sequence variants (ASVs) was less than 1% for most mammalian skin samples and did not exceed 2% for any samples. Although several computer keyboard microbial profiles were dominated by *Nitrososphaeria* sequences, all other skin microbiome data sets tested were primarily composed of sequences affiliated with *Methanobacteriota* and *Halobacteriota* phyla. Our findings revise downward recent estimates of human skin archaeal distributions and relative abundances, especially those affiliated with the *Nitrososphaeria*, reflecting a limited and infrequent archaeal presence within the mammalian skin microbiome.

**IMPORTANCE** The current state of research on mammalian skin-associated archaea is limited, with the few papers focusing on potential skin archaeal communities often in disagreement with each other. As such, there is no consensus on the prevalence or taxonomic composition of archaea on mammalian skin. Mammalian skin health is in part influenced by its complex microbiota and consortium of bacteria and potential archaea. Without a clear foundational analysis and characterization of the mammalian skin archaeome, it will be difficult for future research to explore the potential impact of skin-associated archaea on skin health and function. The current work provides a much-needed analysis of the mammalian skin archaeome and contributes to building a foundation from which further discussion and exploration of the skin archaeome might continue.

## INTRODUCTION

Mammalian skin, which includes skin of both humans and nonhuman mammals, hosts spatially and temporally diverse microbial communities due to extensive chemical and physical variability. Skin topography and epithelial cell type (1), underlying vasculature and endocrine system physiology (2), moisture and oil content (3), and pheromones (4) can all influence microbial colonization and establishment, and these vary according to mammalian host and body site. For instance, sebaceous and apocrine gland secretions create local anoxic areas that provide metabolic substrates for microbial growth (3, 5). Human apocrine glands are located primarily in the armpits (i.e., axillae), whereas rhesus monkeys and baboons have a more diffuse apocrine system (6). The microbial communities that develop according to these physiological differences, primarily represented by *Actinobacteria*, *Bacteroidetes*, *Firmicutes*, and *Proteobacteria* (7), have direct and measurable impacts on host health. For humans, shifts away from a “normal” microbial community composition are associated with eczema (8, 9) and psoriasis (10). Similar links between mammalian dysbiosis and disease have been reported for dogs (11, 12), bovines (13), and camels (14). Therefore, the interconnectivity between mammalian skin physiology, host health, and skin microbiota underscores the importance of elucidating factors that control the diversity and composition of skin-associated microbiomes.

The microbiota of mammals are dominated by bacteria (3, 15–18), although high-throughput sequencing approaches have captured archaeal signatures as well. Early research exploring archaea in mammals focused primarily on the gastrointestinal tract, where methanogenic *Methanobacteriota* (formerly *Euryarchaeota* [19]) members were first detected (20–23). In this context, archaeal communities have been characterized sufficiently to predict mutualistic contributions to host metabolism (24, 25), as well being implicated in disease etiology (26, 27). Archaea are now widely accepted as members of the gut and mucosal microbiota of mammals and more recently have been reported within human breast milk (28). In contrast to many studies of gut-associated microbiota, the mammalian skin “archaeome” is poorly characterized, to the extent that archaea have often been excluded from comprehensive skin microbiome reviews due to insufficient data (3, 29, 30). However, in the last half decade, archaea have been reported as members of skin microbiota of multiple individuals and body locations (24, 31–33). The limited existing research on the skin archaeome demonstrates the need for additional study of mammalian skin and its associated archaeal populations in this emerging field of study.

To our knowledge, Probst and colleagues were the first to demonstrate that archaea can be detected as representatives of human skin microbiota (34). By sampling the torsos of 13 individuals, their study using archaeon-targeting methods estimated that human skin harbors an average archaeal relative abundance of 0.6%, with proportions as high as 4.2% of the total microbial community (34). A subsequent study also used an archaeon-targeted approach to detect an average human skin archaeal community of 1.1% for ages 1 to 11 years, 0.2% for ages 12 to 60, and 4.7% for ages 61 to 75, with a maximum of up to 10.4%. The archaeal communities detected were dominated by putative ammonia-oxidizing archaea (AOA) of the class *Nitrososphaeria* (previously phyla *Crenarchaeota*/*Thaumarchaeota*, now *Thermoproteota*) (32). In contrast, studies using universal prokaryotic (i.e., *Bacteria* and *Archaea*) detection methods suggest a limited skin-associated archaeal community. Extensive skin sampling of cohabitating couples revealed average archaeal sequence relative abundances of less than 0.5%, with archaea detected only from a few samples (16). A human skin study evaluating the impact of polycyclic aromatic hydrocarbon pollutants observed similarly low archaeal relative abundances of less than 0.01% (35). Furthermore, a large-scale human metagenome survey revealed that archaea accounted for approximately 1% of all human metagenome sequences, with the majority of these sequences being affiliated with the *Methanobacteriota* phylum and obtained from gut or mucosal membranes (17). A large study consisting of 589 mammalian skin swab samples concluded that less than 0.1% of all sequences were associated with archaea, with most sequences affiliated with the *Methanobacteriota* and *Halobacteriota* phyla (36). More recently, a shotgun metagenome study investigating udder cleft dermatitis on dairy cows observed archaeon-associated reads nearing 7% relative abundance, represented primarily by *Methanobacteriota* (37).

Skin-associated surfaces in built environments, such as keyboards and door handles, can potentially act as an extension of the skin environment through high frequency contact and deposition of skin microorganisms. Both keyboard and phone microbiomes are influenced by the corresponding finger microbiomes of their users (38, 39), and keyboard microbiomes reflect the skin of their users such that they can be used to identify their specific user (38). From a survey of campus door handle microbiomes, the results revealed that door handles produced microbial profiles that were more similar to skin than to soil or other external environments (40). Archaeal taxa detected on these handles were primarily affiliated with the *Methanobacteriota* and *Halobacteriota* phyla, although archaea were detected at a low relative abundance of less than 0.01% of all amplicon sequences (40). Thus, profiling the archaeome of skin-associated surfaces will enable a better understanding of the detected skin-associated archaea and their allochthonous or autochthonous origins.

Here, to help further address archaeal diversity and relative abundance on mammalian skin, we explored archaeal sequences associated with skin and skin-associated environments using previously published data from human skin (16), nonhuman mammalian skin (36), and door handles (40), as well as newly generated fingertip and keyboard sample data. Using multiple primer sets for a subset of samples, this study evaluated 16S rRNA gene amplicon sequence profiles from 1,058 skin samples (i.e., 458 human and 600 nonhuman mammalian) and 630 skin-associated samples (i.e., 240 keyboard and 390 door handles), for a total of 1,688 sample profiles. We demonstrate infrequently detected presence and low archaeal relative abundance on skin and skin-associated surfaces, with only a few exceptions. When detected, mammalian skin-associated 16S rRNA sequences that affiliated with archaea were primarily assigned to the *Methanobacteriota* and *Halobacteriota* phyla; putative AOA from the *Nitrososphaeria* were largely undetectable.

## RESULTS

This study evaluated the archaeal distributions within eight 16S rRNA gene amplicon data sets (Table 1; see Data Set S1 at https://figshare.com/articles/dataset/Supplemental_dataset_S1_xlsx/14248580). Four were collected from previously published data (“nonhuman mammalian skin,” “human skin,” “door handles,” and “Roche 454”), two were from newly obtained samples (“keyboard” and “fingers”), one represented a subset of the mammalian and human skin data, with 92 samples prioritized by archaeal presence (“Pro341F/Pro805R”), and another was composed of the same sample subset that was processed again for sequencing by using an alternate universal prokaryotic primer set (“515F-Y/926R”). All primers used to generate amplicon data included in this study were tested *in silico* for primer coverage. The Pro341F/Pro805R and 515F-Y/926R comparison data sets were generated to test for possible primer bias against archaeal 16S rRNA gene sequences in previous data. Overall, coverage of the domain *Archaea* by the universal prokaryotic primers (i.e., Pro341F/Pro805R and 515F-Y/926R) was extensive (Table 2). Both primers had greater than 65% coverage of *Archaea* at zero mismatches permitted, although *Nitrososphaeria* coverage was limited depending on the primer set (515F-Y/926R, 83%, and Pro341F/Pro805R, 16%). However, with only one mismatch permitted (not proximal to the 3′ end), both primers had greater than 85% coverage for both *Archaea* and *Nitrososphaeria*. Conversely, the archaeon-specific primers (i.e., 344af/517ur and 344af/915ar) that were used to generate Roche 454 data had below 50% coverage for *Archaea* at zero mismatches permitted but increased to 65% to 71% for one non-3′-end mismatch. Coverage of *Nitrososphaeria* with these archaeon-specific primers remained below 40% for both zero and one mismatch permitted.

**TABLE 1:** Summary of skin and skin-associated data sets

Study (reference)	No. of samples	Sample type(s)	Method	Primers	No. of reads		PCR cycles
					Total^*a*^	Avg/sample ± SD	
Nonhuman mammalian skin (36)	546	Skin	Swab	Pro341F/Pro805R	5,238,782	9,594 ± 7,840	40^*b*^
Human skin (16)	340	Skin	Swab	Pro341F/Pro805R	7,631,068	22,444 ± 17,611	40^*b*^
Pro341F/Pro805R^*c*^	92	Human and mammalian skin	Swab	Pro341F/Pro805R	1,000,284	10,872 ± 9,490	40^*b*^
515F-Y/926R	92	Human and mammalian skin	Swab	515F-Y/926R	573,461	6,233 ± 4,803	40
Fingers	80	Fingers	Swab	515F-Y/926R	1,722,646	21,533 ± 12,498	45
Keyboard	240	Keyboards	Swab	515F-Y/926R	5,972,034	24,883 ± 15,353	45
Door handles (40)	390	Door handles	Swab	Pro341F/Pro805R	4,859,381	12,252 ± 5,359	35
Roche 454 (32)	22	Human skin	Swab	344af/917ar	54,554	2,479 ± 5,466	35

a The total reads represent the number of reads output following the merge-denoise and chimera removal steps in the workflow.

b Nested PCR was performed by first amplifying the V3-V4 hypervariable region of the 16S rRNA gene for 25 cycles, followed by 15 cycles for Illumina adapter ligation.

c The data presented for Pro341F/Pro805R are a subset of data from the human and mammalian skin data sets.

**TABLE 2:** Comparison of the bacterial and archaeal coverage of universal prokaryotic and archaeon-specific primers

Primer pair	% coverage with^*a*^:					
	Zero mismatches			One mismatch		
	*Bacteria*	*Archaea*	*Nitrososphaeria*	*Bacteria*	*Archaea*	*Nitrososphaeria*
515F-Y/926R	84.5	81.0	82.5	90.7	88.8	86.8
Pro341F/Pro805R	82.6	68.2	16.2	88.4	86.3	92.2
344af/517ur	0	30.7	15.1	0	71.2	36.8
344af/915ar	0	44.6	12.9	0	65.4	32.0

a Each primer set was tested *in silico* using SILVA TestPrime version 1.0 (81) using the SSU 138 database. Primers were tested without allowing mismatches (“zero mismatches”) or allowing a single mismatch provided that it did not occur within five bases proximal to the 3′ end (“one mismatch”).

**FIG 1:**
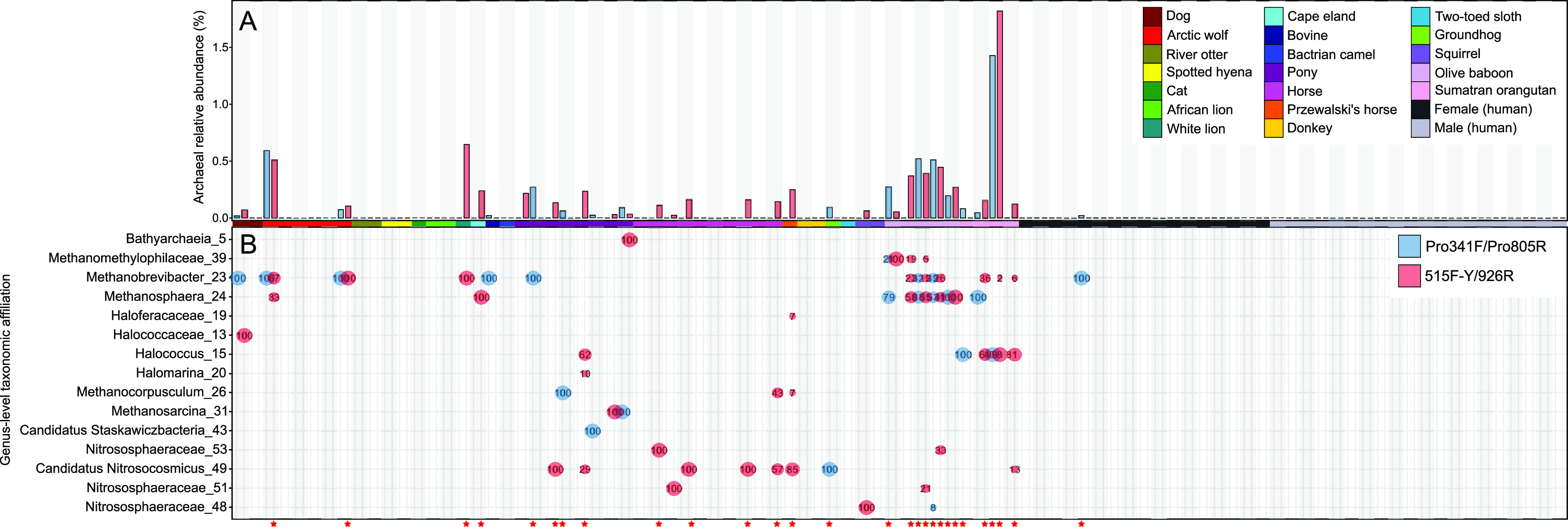
Comparison of the relative abundances (A) and ASVs (B) of archaea on human and nonhuman mammalian skin. Samples were sequenced using primer pairs 515F-Y/926R and Pro341F/Pro805R and their relative abundances compared, with pairs indicated with alternating gray/white background bars. The relative abundances of the ASVs are indicated by both percentage and size of the point. Red stars at the bottom indicate samples with more than five archaeal reads; all unmarked samples had four or fewer reads.

Archaeal relative abundances (Fig. 1A) and amplicon sequence variant (ASV) profiles (Fig. 1B) were similar based on profiles generated with the 515F-Y/926 and Pro341F/Pro805R primer pairs. Archaea were detected in 26 and 18 of the 92 samples (28% and 20% of samples) for the 515F-Y/926R and Pro341F/Pro805R primer pairs, respectively (Table 3), with average archaeon-associated read counts of 25 ± 49 (mean ± standard deviation) and 14 ± 19 (see Data Set S1 at https://figshare.com/articles/dataset/Supplemental_dataset_S1_xlsx/14248580). Of the 20 samples with detectable archaea generated with only one of the two primer pairs, archaeal relative abundances did not exceed 0.3%, except for a single mammalian skin sample with a relative abundance of 0.65%. Archaea were largely undetected in samples from human skin, regardless of the primer pair used. For samples with archaea detected using both primer sets, archaeon-associated ASV profiles were similar (Fig. 1B). Samples with a low number of reads varied the most from their paired samples, whereas pairs with higher read counts were highly similar (Fig. 1B). The 16S rRNA gene profiles generated using the 515F-Y/926R primer set typically contained additional archaeon-associated ASVs (Fig. 1B), albeit in lower abundance than the dominant ASVs common to paired samples analyzed with both primer sets.

**TABLE 3:** Summary of archaeal reads and ASVs of the skin and skin-associated environment

Data set	Total no. of samples	No. of:		Total no. of ASVs	No. of:		% archaeal abundance	
		Samples with archaea detected	Archaeal ASVs		Archaeal reads	Bacterial reads	Relative^*a*^	Maximum
Nonhuman mammalian skin	546	194	121	24,306	4,179	5,238,782	0.079	1.510
Human skin	340	20	25	9,613	176	7,631,068	0.002	0.264
Pro341F/Pro805R^*b*^	92	18	20	5,399	260	1,000,284	0.026	1.429
515F-Y/926R	92	26	31	5,827	668	573,461	0.116	1.820
Fingers	80	2	2	1,604	193	1,722,646	0.011	0.418
Keyboard	240	15	33	6,623	935	5,972,034	0.015	1.116
Door handles	392	39	28	13,546	354	4,859,381	0.007	0.522

a The relative abundances of archaea were calculated using all reads for each data set.

b The data presented for Pro341F/Pro805R are a subset of data from the mammalian and human skin data sets.

**FIG 2:**
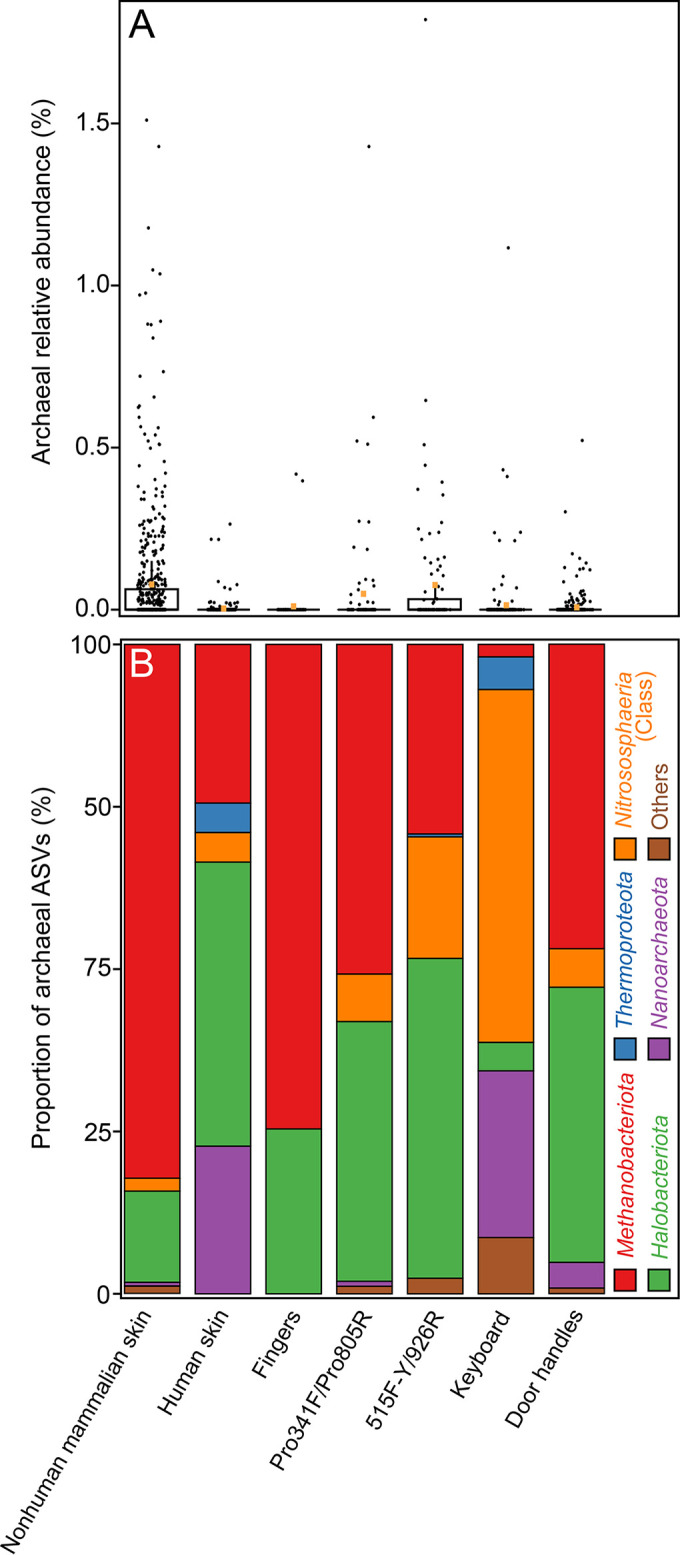
(A) Archaeal 16S rRNA gene relative abundances. The relative abundances of archaeal sequences were calculated by dividing the number of sequences affiliated with archaeal ASVs by the total number of sequences for each sample. Relative abundance averages for all samples in each data set are indicated by orange squares within the boxplot. (B) The taxonomic proportions of the archaeome of the skin and skin-associated surfaces are separated by phylum or class. Archaeal taxonomic proportions include archaeal 16S rRNA gene reads only and represent the proportions of archaeal reads belonging to each phylum or class. Class *Nitrososphaeria* was separated from the phylum *Thermoproteota* to highlight putative AOA-associated archaea specifically. The *Thermoproteota* category thus does not contain any *Nitrososphaeria*-associated reads.

Of the eight data sets included in our analysis (Table 3), the subset comprised of mammalian skin samples that were amplified with 515F-Y/926R universal prokaryotic primers had the highest archaeal relative abundances, representing 0.12% ± 0.22% of all reads overall (Fig. 2A). The nonhuman mammalian skin sample set had the second highest archaeal relative abundance (0.08% ± 0.04%) and accounted for the majority of archaeon-positive samples (*n* = 194; 65.5%) within the study, although most samples (*n* = 352; 64.5%) did not contain any detected archaea. Human skin had a very low archaeal relative abundance (2.0 × 10^−3^ ± 0.02%), with only a fraction of samples (5.9%) containing archaeon-associated sequences. The archaeal relative abundance of the finger data set was similarly low (0.01% ± 0.06%) and confined to two samples (2.5% of the data set). The keyboard and door handle data sets were also low (0.02% ± 0.09% and 7.0 × 10^−3^ ± 0.04%). Despite these average relative abundances, most samples from all eight data sets (excluding the previously published Roche 454 data set [*n* = 1,392; 82.5%]) had no detected archaeal reads, despite thousands of reads per sample. Although most samples had no detected archaea, several samples (*n* = 296; 17.5%) contained archaeon-associated sequences, though none exceeded 2% relative abundance. For example, sample 16SOB, which was sourced from an olive baboon and had the highest archaeal relative abundance of any mammalian skin sample, had relative abundances of 1.51% with primers Pro341F/Pro805R and 1.82% with primers 515F-Y/926R. The only other sample with an archaeal relative abundance greater than 1% belonged to the keyboard data set, at 1.12%, sourced from a female participant between 20 and 29 years of age. Together, the data generated with both universal primer sets yielded only a small proportion of archaeon-positive skin or skin-associated-surface samples.

The taxonomic distributions of archaea (Fig. 2B) and the numbers of archaeal ASVs varied among data sets and were dominated primarily by only a few phyla. Nonhuman mammalian skin was dominated by *Methanobacteriota* sequences, which constituted 82.2% of all archaeal reads. *Halobacteriota* and *Nitrososphaeria* were the next most prevalent phyla, corresponding to 14.0% and 1.99% of archaeal reads, respectively. Conversely, the human skin archaeome was comprised of *Halobacteriota* (43.8% of archaeal reads), *Methanobacteriota* (24.4% of archaeal reads), and *Nanoarchaeota* (22.7% of archaeal reads). Similar to the mammalian skin archaeome, *Nitrososphaeria* represented a small proportion of archaeon sequences (4.6% of archaeal reads). The finger swab data set contained archaea belonging to only the *Methanobacteriota* and *Halobacteriota* phyla, at 74.6% and 25.4% of archaeal reads, respectively. The Pro341F/Pro805R and 515F-Y/926R data sets, representing a subset of all human and mammalian skin samples, both revealed high proportions of *Methanobacteriota* and *Halobacteriota*. Overall, within all archaeal sequences from the subset of all mammals and humans, the 515F-Y/926R data set showed an increase in the proportion of *Nitrososphaeria* (18.7% of archaeal reads) compared to the Pro341F/Pro805R data set (7.3% of archaeal reads). Door handles were dominated by *Methanobacteriota* (46.9% of archaeal reads) and *Halobacteriota* (42.4% of archaeal reads). In contrast, sampled computer keyboards were dominated by *Nitrososphaeria* (54.3% of archaeal reads) and *Nanoarchaeota* (25.7% of archaeal reads).

**FIG 3:**
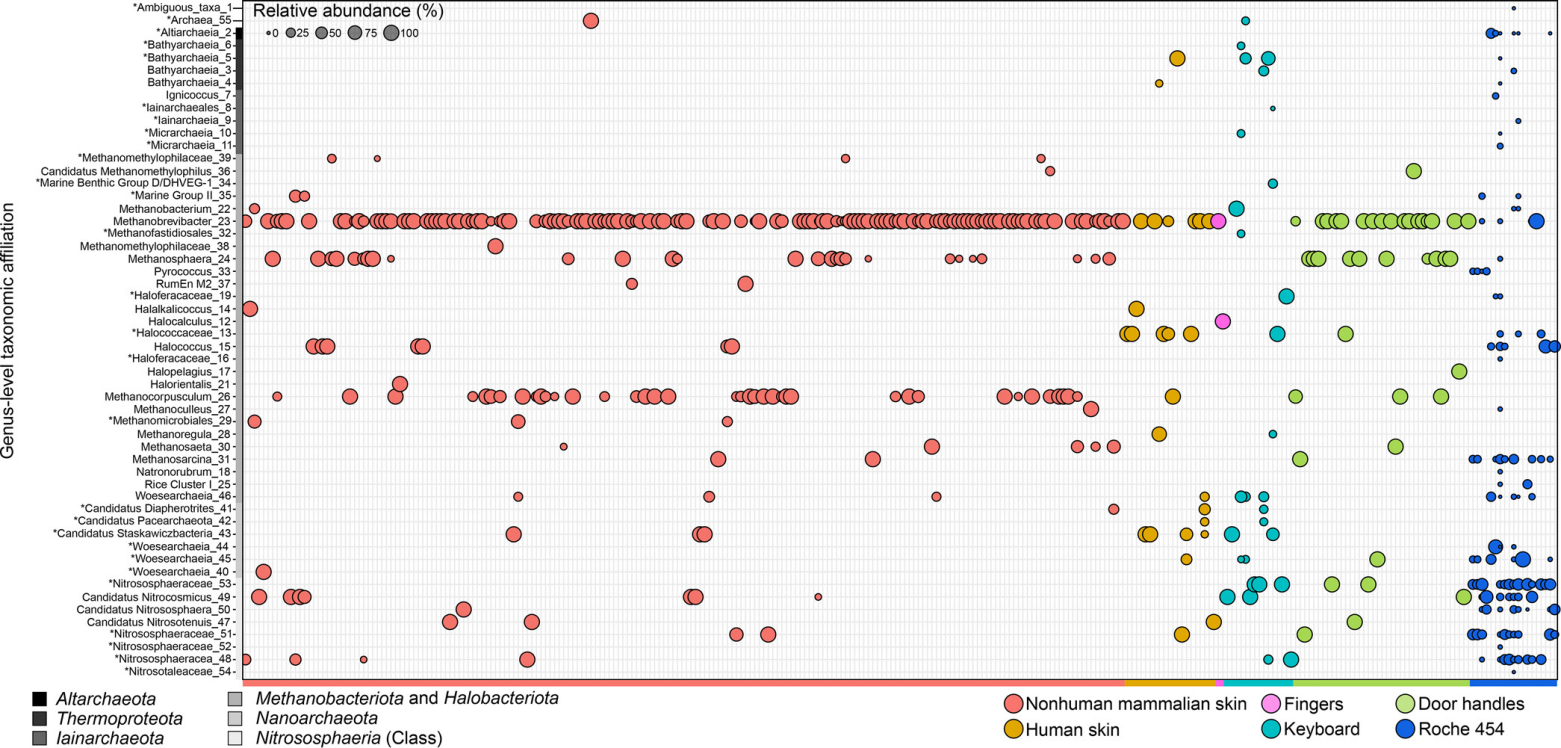
Distribution of all archaeal genera across skin and skin-associated surfaces. The ASV table was collapsed to the genus level and then filtered for archaeal taxa and contains any sample with a nonzero number of archaeal 16S rRNA gene reads. The sizes of the bubbles represent the relative abundances of the genera with respect to the total number of archaeal 16S rRNA gene reads within a sample. Archaeal ASVs not resolved to the genus level were collapsed to their most resolved taxonomic level and are indicated with asterisks, and they are referred to as “genera” in Results and Discussion. A human skin data set (32) was included for comparison.

Analysis of all archaeal genera present within each data set (including the Roche 454 data set) showed some overlap, with 31 of 54 genera observed in at least two data sets (Fig. 3). The remaining 23 genera were unique to a single data set. These unique genera span the archaeal DPANN and TACK superphyla and include ASVs present at various relative abundances. Within the *Methanobacteriota*, *Methanobrevibacter* was the most common genus, found in nearly all samples, and it was also present in the previously published Roche 454 human skin amplicon data set (32). Another genus affiliated with the *Methanobacteriota* is *Methanosphaera*, which was found associated with several samples, either alone or in combination with *Methanobrevibacter* sequences. Various *Halobacteriota* were present, with *Halococcus*/*Halococcaceae* and *Methanocorpusculum* found within several mammalian skin samples, including in the Roche 454 data set. Although the *Halobacteriota* (excluding *Methanocorpusculum*) were not prevalent across samples within a data set, they accounted for a considerable proportion of all archaeal reads (Fig. 2B). The AOA-associated *Nitrososphaeria* were present but were rare and detected in only 79 of 1,710 samples (4.6%) included in this analysis (Roche 454 included). Of those 79, 49 (62.0%) belonged to the Roche 454 data set. The nonhuman mammalian skin and Roche 454 data sets shared the same *Nitrososphaeria*-classified genera, except for one ASV that could not be resolved to the genus level (i.e., *“*Nitrosotaleaceae_54”), which was associated exclusively with the Roche 454 data set (Fig. 3). Among samples from nonhuman mammalian skin, AOA-associated genera were found on the skin of several mammals (i.e., olive baboon, squirrel, donkey, dog, groundhog, cheetah, and Asian elephant). The genus *Nitrosocosmicus* was absent from human skin data sets, although ASVs associated with this genus were present in all other data sets (i.e., nonhuman mammalian skin, keyboards, fingers, Roche 454, and door handles) and was one of the more common archaeal genera detected.

**FIG 4:**
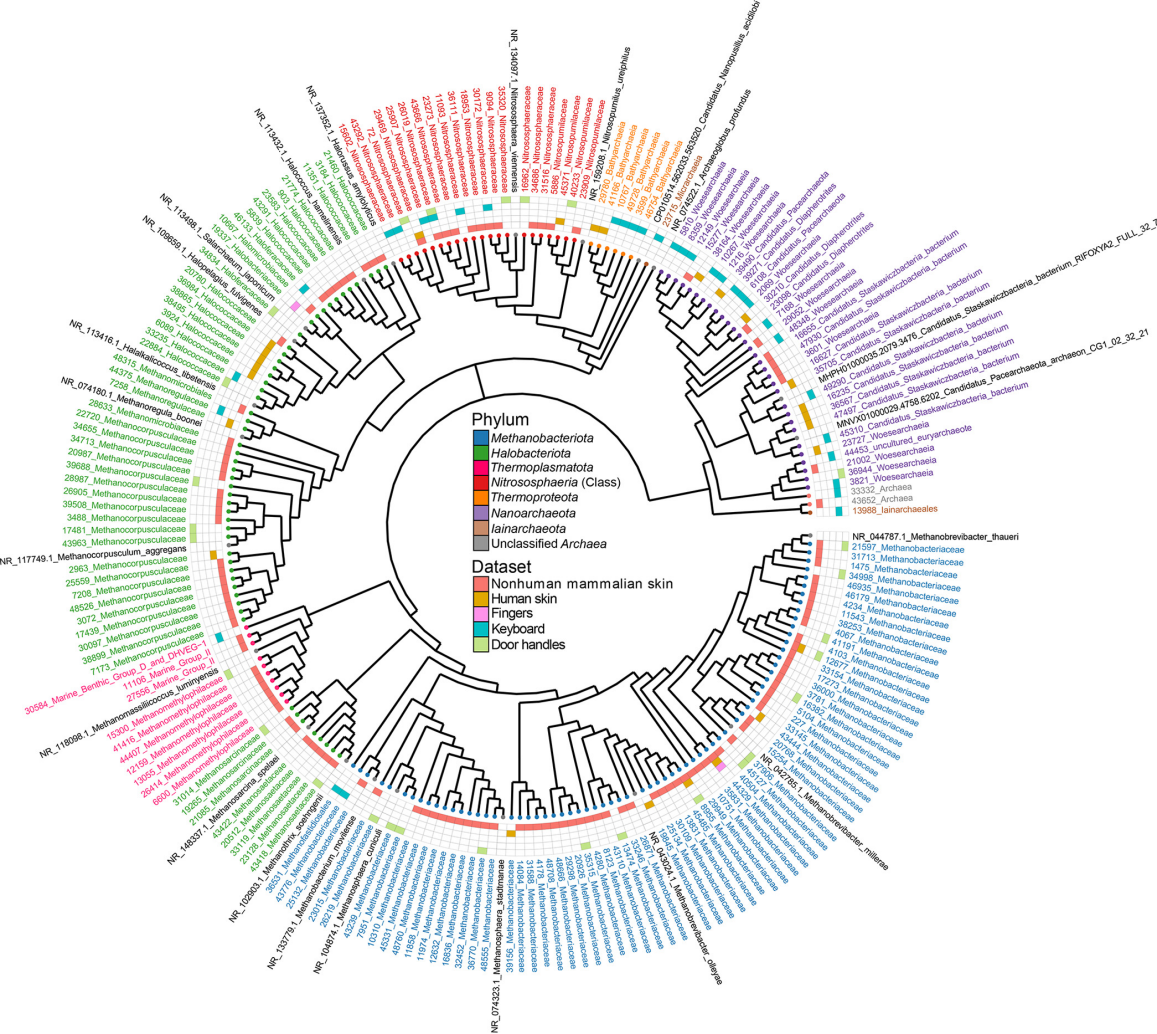
The prevalences and overlaps of archaeal ASVs on skin and skin-associated surfaces. The tree contains all archaeal ASVs from each data set. ASVs in black are 16S rRNA gene reference sequences retrieved from the NCBI and SILVA databases, whereas the remaining ASVs are colored according to their respective class or phylum. Because not all ASVs were resolved to the species or genus level, all ASVs were renamed to a family level for consistency. ASV overlaps between data sets are indicated through the heatmap squares. The maximum-likelihood tree was constructed using a GTR +G +I model with bootstrap support of 1,000.

An analysis of 193 archaeal ASVs associated with new and previously published data sets analyzed in this study (Roche 454 excluded) revealed that the archaeal ASVs detected were differentially distributed between data sets and that the overlap between data sets was limited (Fig. 4). The nonhuman mammalian data set contained 57 of the 69 (82.6%) *Methanobacteriota* ASVs detected; 49 (71.0%) of those were unique to the data set. The remaining eight ASVs were observed in two or more data sets, including three *Methanobacteriota* ASVs that were present in three data sets (44329_Methanobacteriaceae, 12677_Methanobacteriaceae, and 8955_Methanobacteriaceae). The *Halobacteriota* were similarly dominant, with 35 of 52 (67.3%) total ASVs being unique to non-human mammalian skin. Only a single *Halobacteriota* ASV, 43418_Methanosaetaceae, was shared between more than one data set, and it belonged to both nonhuman mammalian skin and human skin samples. The 21 *Nitrososphaeria* ASVs originated solely from nonhuman mammalian skin (*n* = 9; 42.9%), keyboard (*n* = 4; 19.0%), door handle (*n* = 4; 19.0%), and human skin (*n* = 1; 4.8%) data sets, with *Nitrososphaeria* absent from the separate finger data set. The remaining three (14.2%) *Nitrososphaeria* ASVs were shared between data sets, with two of the ASVs being shared between two data sets and a single ASV, “26019_Nitrososphaeraceae,” shared among three data sets: nonhuman mammalian skin, keyboards, and door handles. The ASVs associated with *Thermoproteota*, *Iainarchaeota*, and *Nanoarchaeota* represented the remaining 51 (26.4%) ASVs and were observed more broadly within multiple data sets but had no overlap among data sets.

## DISCUSSION

### Archaea on mammalian skin are rare and uncommon.

This amplicon-based assessment of skin and skin-associated microbiota revealed an infrequent and low-abundance distribution of archaea among all 1,688 samples from skin and skin-associated amplicon data sets. The limited detection of archaea on mammalian skin suggests that archaeal communities are relatively rare, of low relative abundance, and below common detection limits when using PCR with universal prokaryotic primers and high-throughput sequencing. The disparity between human skin and nonhuman mammalian skin is expected considering the large differences in their local environments and hygiene practices. The majority of nonhuman mammalian skin samples contained no detectable archaeal sequences, and no sample exceeded 2%. Given differences in skin physiology, living conditions, and geographic origins of the hosts, it was not unexpected to observe large variations in archaeal distributions among mammalian skin samples, particularly within samples where archaea were detected. For example, although human and nonhuman mammalian skin samples both contained sequences associated with *Methanobrevibacter*, nonhuman mammalian skin samples had much greater comparable relative abundance and sample prevalence. Although *Methanobrevibacter* taxa are typically host associated within the gut (41, 42), they are suggested to be distributed in soil (43) and water (44), and previous results suggest that a transient environmental skin layer is common for mammalian hosts (36). It is perhaps more likely that gut-associated *Methanobrevibacter* are instead being deposited into the environment, via animal feces, which are then taken up onto the skin. The AOA are abundant members of soil (and other environments), where they contribute to the biogeochemical cycling of nitrogen (45), and they have also found been sporadically and at low relative abundance among mammalian skin samples. *Nitrososphaeria*-associated reads comprised only a small portion of the total archaeal reads in nonhuman mammalian skin (1.9%) and human skin (4.5%) samples and are likely derived from environmental sources (45, 46). Terrestrial and semiaquatic nonhuman mammals in close contact with their respective environments and debris (e.g., feces and urine) and without the routine hygiene practices of humans are perhaps more likely to have skin communities containing allochthonous archaea from environmental reservoirs. The 12 shared archaeon-associated ASVs in all data sets were associated with the *Methanobacteriota*, *Nitrososphaeria*, and *Halobacteriota*. These taxa have been observed in environmental (43, 44) or commercial (e.g., food and cosmetics) (47) samples, which might explain their ubiquity across data sets.

The samples from skin-associated surfaces in built environments were equally absent of detected archaea, with the majority of keyboard (93.8%) and door handle (90.0%) samples containing no detected archaea. The keyboard and door handle built environments have different chemical and physical environments from skin but, via frequent human contact, can develop skin-like microenvironments and microbial communities (38, 39). The few archaeal sequences present on door handles were associated with archaea in a pattern similar to that observed in samples from human and nonhuman mammalian skin: primarily *Methanobacteriota* and *Halobacteriota* and likely derived from environmental reservoirs as suggested elsewhere (40). Additionally, food and cosmetic reservoirs for *Halobacteriota*-associated genera have been identified (47) and might serve as an explanation for the observed archaeal sequences. However, although only representing less than 0.02% of the total reads, the archaeal community proportions observed on keyboards were markedly different from any other analyzed data set within this study. Instead of a dominance of *Methanobacteriota*, as seen in other data sets analyzed here, the AOA-associated class *Nitrososphaeria* collectively contributed more than 50% of archaeal reads. Most of these reads (>90%) could be attributed to two individuals: a female between the ages of 20 and 29, and a female of more than 60 years of age. The detected *Nitrososphaeria* may not be entirely representative of the typical keyboard microbiota, given that *Nitrososphaeria*-associated reads were absent from most other keyboard sample data. Instead, these sequences might be attributed to individual differences in the keyboard users themselves or their activity habits (e.g., eating multiple meals at the keyboard or their local environments).

Metagenomic studies focusing on skin-associated archaea are rare, making assessment of the skin archaeome difficult. Only a small number of studies address skin archaea directly with archaeon-targeted approaches (31–34). All other skin-associated archaeal data are produced from holistic skin microbiome studies utilizing universal prokaryotic primers and 16S rRNA gene amplicon sequencing (16, 35, 36) or metagenome-assembled genomes (17, 37, 48), where attention to skin-associated archaea is minimized due to their low abundance. The data presented on the rarity of skin-associated archaea in the current work, along with the comparisons made to existing skin archaeon literature, represent nearly the entirety of skin archaeome research. However, skin microbiome profiling can be impacted by several factors, which could affect the profiling of the skin archaeome. Our current study samples only the outermost portion of the epidermis (i.e., stratum corneum), and previous research shows that the lower epidermis and subepidermal areas of the skin also harbor microbial communities (49). As well, we analyze samples from a human population specific to a region in North America, even though regionality and race may impact skin microbial communities (50). Furthermore, individual hygiene habits, such as deodorant use (51) and showering (52), may impact microbial community composition. Additional research would be needed to fully address myriad factors that may influence the abundances and distributions of archaea within the skin microbiomes of humans and other mammals.

### Implications for previous studies of human skin archaea.

Although the same genera were shared between this study and those from a Roche 454 data set (e.g., Fig. 3), our data suggest much lower archaeal relative abundances for human skin samples than have been estimated previously using quantitative PCR (qPCR) archaeal and bacterial 16S rRNA proportions (32, 34). The primary causes for these differences may be primer and method specific. Successful detection of archaeal sequences is dependent on using primers that include adequate coverage of the domain *Archaea*. In the current study, we used two universal prokaryotic primer pairs that, at least *in silico*, have extensive coverage of the archaeal and bacterial domains (Table 2). The same DNA extraction method and 515F-Y/926R prokaryotic primers were recently used to detect archaeon-associated ASVs of the AOA *Nitrosocosmicus* genus in wastewater treatment plants (53), with very high agreement between archaeal abundances (∼1%) observed among amplicon-based, qPCR, metagenomic, and fluorescent *in situ* hybridization (FISH) methods (53, 54). Additionally, an evaluation of several common universal prokaryotic primers concluded that 515F-Y/926R was the optimal primer pair for detecting archaea in marine ecosystems (55). We have also demonstrated with direct comparisons that archaeal detection is similar between the two primer pairs. Universal prokaryotic primers allow archaea to be coamplified alongside bacteria, providing a sample-by-sample characterization of archaeal relative abundances. Although this provides community context, it has been suggested that coamplification of bacteria and archaea could introduce bias against archaea because of low-template exclusion (56, 57). Alternatively, studies by Probst et al. (34) and Moissl-Eichinger et al. (32) estimated absolute abundances based on qPCR using archaeon-specific 344af/517ur primers. Taxonomic information was generated using the 344af/915ar primers separately for 16S rRNA gene sequencing (32). Although community context is lost, because of the exclusion of bacterial 16S rRNA genes during amplification, the archaeon-specific primers could amplify rare archaeal 16S rRNA gene sequences that otherwise might be outcompeted by the bacterial 16S rRNA template.

Extraction bias and uncertainty associated with qPCR amplicon specificity and the capacity for the 344af/517ur primers to unintentionally amplify the bacterial template are concerns. Using biased extraction methods that do not account for the physiological differences between bacterial and archaeal cell structures (e.g., S-layer and methanochondroitin) (58) has been suggested as a reason for low archaeal abundances observed on skin (32). The PowerSoil kits used in the current study do not impact archaeal diversity (59), and the additional pretreatment steps we have included should minimize bias. However, postextraction nonspecific amplification of bacterial sequences could result in an overestimation of archaeal abundances during qPCR. Justification for this concern is found in the 344af/915ar sequencing data of Moissl-Eichinger et al. (32). Although this primer pair was used only to generate archaeal taxonomic data, several samples contained bacterial sequence contamination, ranging from 10% to 50% of all reads. The primers used for qPCR share the same archaeon-specific forward primer that was used in sequencing (344af), but also a universal prokaryotic 517ur reverse primer. Although the primer pair shows very low bacterial coverage *in silico* (33), the less specific 344af/517ur pair might have amplified bacterial 16S rRNA gene sequences. If so, the coamplification of bacterial sequences during archaeal qPCR quantification could be undetected, and both the bacterial and archaeal products would be unintentionally combined and used to quantify archaea. The resulting abundances would be overestimated, and this could explain the high reported archaeal abundances. If not attributed to regional differences (e.g., cultural or regional differences in skin communities, surrounding environments, or lifestyles), the relatively high skin archaeal abundances reported previously could be attributed to methodological bias.

Although PCR and primer biases that confound archaeal amplification may exist, we maintain that if archaea are routinely abundant on mammalian skin, the large sample size presented in our study would have revealed this to be the case. At the least, it would be expected that the number of samples with detected archaeal sequences would be higher than currently presented, regardless of abundance. Notwithstanding, the differences in quantitation method do not explain the taxonomic profile differences observed in the literature. Whereas we show considerable *Methanobacteriota* and *Halobacteriota* dominance, some prior literature suggests that *Nitrososphaeria* were the most abundant taxa (32, 34). If *Nitrososphaeria* were a common skin community member, we expect that the increased archaeal coverage and large sample size of the current study would reveal more *Nitrososphaeria* prevalence or relative abundance among samples. Indeed, this is the case for the keyboard microbiomes, where more than 50% of the archaeal sequences belong to *Nitrososphaeria*.

Other than potential methodological bias, the differences between our amplicon data and previous qPCR or Roche 454 data might be explained by factors that vary due to the geographical origins of human subjects (such as personal hygiene, activity type, or geographic region in Canada versus Northern Europe) or some other as-yet-unidentified factor. For example, the gut microbiomes of individuals in areas of Europe contain more *Methanobacteriota* sequences than the gut microbiomes of individuals in North America (60). Although there is extensive literature that attempts to define a core skin panmicrobiome (7, 18, 61), archaea are often absent from these studies and reviews. If archaea are established members of the skin microbiome, then the potential regional (e.g., forest versus grassland) (62) and population differences that drive the observed variations in diversity and abundance could have a considerable and currently poorly quantified impact on archaeal abundance on human skin.

Previous research of human skin archaea also used FISH (34) and Fourier transform infrared focal plane array (FTIR-FPA) hyperspectral imaging (32). Both methods suggested the presence of archaea on human skin, although they do not offer quantitative information and are not linked to any other sample data (32). Although FISH was intended to verify the presence of archaea on human skin (34), the ARC915 probe has extensive coverage for several archaeal phyla, including *Methanobacteriota* and *Halobacteriota*. Thus, cells observed with this method should be interpreted cautiously and should be confirmed in the future with multiple probes and samples to help verify the identify of FISH-positive cells.

### Skin as a potential habitat for archaea.

Although the data presented suggest that archaeal occurrences on human skin are infrequent and that occurrences on mammalian skin are comparatively more common though still rare, the presence of archaeon-associated sequences in some samples may nonetheless be relevant to host habitat and health. Archaeon-associated genera and ASVs shared among data sets could provide insight into common archaeal detection, colonization, or contamination of the skin and skin-associated surfaces. For example, many of the prominent archaeal genera observed in our data sets were observed in the Roche 454 human skin data set produced in a separate laboratory, with different methods, and in a distant geographic location (32). The detection of these shared archaea, despite differences in detection techniques, might suggest the existence of a more “core” archaeome comprised of ubiquitous soil-associated archaea, albeit at very low abundance. The one *Nitrososphaeria-* and three *Methanobacteriota*-associated ASVs that overlapped concomitantly with human and nonhuman mammalian skin could represent archaeal skin ecotypes that might be more adapted to the physical and chemical environment of mammalian skin.

With such low archaeal abundances detected in our study, it is difficult to make conclusions about whether the skin archaea represent autochthonous populations or allochthonous environmental contaminants. However, there are several features of mammalian skin that might allow archaeal populations to establish. For example, mutualistic relationships between acetogenic bacteria and methanogenic *Methanobacteriota* archaea have been documented (63), and it could be that the same community interactions are occurring on the skin, although it would require localized zones of anoxia. These anoxic zones could exist on the skin as a natural result of sebaceous and apocrine secretion onto the skin surface (3, 5). Although sequences associated with acetogenic bacteria and methanogenic archaea were found within the same samples (36), it is uncertain whether such syntrophic interactions occur.

Several halophilic genera were detected on mammalian skin, in addition to other skin-associated environments. Although mammalian skin might be a suitable environment for *Halobacteriota* archaea, due to its variations in salinity compared to the salinities of other surface environments, these microorganisms are more likely to be contamination from food and skin care products due to their frequent consumption and use by humans (47). However, halophilic archaea have been previously reported in the human oral and intestinal tracts (22, 64), which could provide a route through which they might colonize skin or renew their populations.

Putative AOA-associated genera were detected within a relatively small number of mammalian skin samples. Nonhuman mammalian species could be exposed to ammonia-containing substrates from their environment (e.g., animal waste) that could provide an energy source to AOA established on skin surfaces. Mammalian skin might also passively diffuse ammonia (65, 66) or excrete it in sweat (67), and there is the potential for localized skin diseases to increase ammonia production (68). Furthermore, common shampoos and hand soaps often contain ammonia derivatives that could facilitate AOA growth. Nonetheless, *Nitrososphaeria*-associated reads made up only a small portion of the total archaeal reads (nonhuman mammalian skin, 1.9%, and human skin, 4.5%). If nonhuman mammalian skin can maintain a suitable environment for AOA metabolism, these archaea appear to represent rare and relatively low-abundance populations.

### Conclusion.

The exploration and microbial profiling of the skin and of skin-associated environments is an integral component to understanding the interactions of the skin microbiome and host health. Here, we provide evidence that archaea are rare and infrequent members of human and nonhuman mammalian skin and skin-associated-surface communities. For samples with detectable archaea, individual host and environmental variations might explain archaeal distributions. Shared ASVs and genera among data sets provide insight into this rare-biosphere skin archaeome, regardless of whether they are autochthonous or allochthonous community members. Overall, we challenge recent literature suggesting an unexpected relative abundance of human skin archaea and suggest that future research using shared samples and validated and standardized methods could be helpful. For example, future skin archaeome characterization using universal prokaryotic primers could benefit from an internal sequencing workflow standard made of both archaeal and bacterial 16S rRNA gene sequence templates (69). Thus far, our amplicon-based data agree with the conclusion of a comprehensive skin microbiome metagenomic assessment: “Archaea were nearly absent on skin” (48).

## MATERIALS AND METHODS

### Sample collection, selection, and processing.

Keyboard and finger swab samples from individuals between the ages of 18 and 70 were collected for this study in accordance with the University of Waterloo Office of Research Ethics (ORE) project 40212. After participants washed their hands, the index and middle fingers on the left and right hands were swabbed for 30 s in a circular motion with a sterile foam swab (Puritan Medical Products, Guilford, ME, USA). Participants were then asked to perform a typing exercise 10 times (“The quick brown fox jumped over the lazy dog 1234567890”). The same fingers were swabbed again using new sterile swabs. Twenty-four keyboard keys per keyboard were selected to capture different usage frequencies based on an analysis with WhatPulse version 2.8.0. Each individual key was swabbed for 30 s with a new sterile swab. All swabs were then stored in applicator tubes in a −20°C freezer. Genomic DNA was extracted from swab samples using the PowerSoil DNA isolation kit (Qiagen, Canada) using the manufacturer’s protocol with minor modifications. Swab tips were removed with a flame-sterilized sterile scalpel, deposited into a PowerSoil bead-beating tube, and incubated at 70°C for 10 min on a rotating holder. The tubes were then subjected to mechanical lysis using a FastPrep 24 agitator (MP Biomedicals, Solon, OH, USA) at 5.5 m/s for 45 s and extracted following the manufacturer’s protocol. The samples were eluted in 10 mM Tris and stored at −20°C prior to PCR amplification and sequencing.

Previously published data from mammalian (36) and human (16) skin studies and a campus door handle survey (40) were compiled here for comparison. The Pro341F/Pro805R V3-V4 primers used in these studies were selected based on their original design for increased archaeal detection (70). To test whether archaeal distributions were influenced by universal prokaryotic primers used in generating results for these earlier studies (Pro341F/Pro805R; V3-V4 regions) (70), we obtained DNA extracted from 38 human and 54 mammalian skin samples (92 samples total) to generate new 16S rRNA gene profiles using additional universal prokaryotic primers as detailed below. Samples included in the primer set comparison were selected based on previously observed archaeal sequences in those samples, such that samples with greater archaeal relative abundances were selected to increase the likelihood of obtaining sequences for direct comparison.

### PCR and sequencing.

The V4-V5 regions of the 16S rRNA gene were amplified from all fingertip swab samples, keyboard swab samples, and the 90-sample subset of human/mammalian skin samples (see above) using universal prokaryotic primers 515F-Y (5′-GTGYCAGCMGCCGCGGTAA-3′) (71) and 926R (5′-CCGYCAATTYMTTTRAGTTT-3′) (72). Both primers were modified to include a 6-base barcode sequence used for identification of amplicons, an adaptor sequence for flow cell binding, and an Illumina primer binding site (73). The PCR was performed in a sterile ISO class 5 HEPA PCR hood, which was cleaned with 70% ethanol before being treated with UV for 15 min prior to use. A PCR master mix was created using UV-treated PCR-grade water, 1× ThermoPol buffer, 0.2 μM forward primer, 0.2 μM reverse primer, 200 μM deoxynucleoside triphosphates (dNTPs), 15 μg bovine serum albumin (BSA), 0.625 units of Hot Start *Taq* DNA polymerase (New England Biolabs, Ipswich, MA, USA), and 1 μl of DNA template in each 25-μl reaction mixture. Positive and negative PCR controls were included, as were extraction kit controls. Amplification was performed using a T100 thermal cycler (Bio-Rad Laboratories, Canada) using the following reaction conditions: 95°C initial denaturation for 3 min and then 40 cycles of 95°C denaturation for 30 s, 55°C annealing for 30 s, and 68°C extension for 1 min, with a final extension at 68°C for 7 min. All PCR amplifications were performed in triplicate and then pooled in equimolar quantities before purifying on a 1% ethidium bromide gel. Amplicons were extracted from the gel and purified using a Wizard SV gel and PCR clean-up system (Promega, Madison, WI, USA). The library was diluted to 8 pM, and 15% PhiX control version 3 (Illumina, Canada) was added prior to sequencing. The 515F-Y/926R samples were sequenced using a 2 × 250 cycle TG MiSeq reagent nanokit version 2 (catalog number MS-103-1003; Illumina) on a MiSeq instrument (Illumina). The keyboard and finger samples were sequenced using a 2 × 250 cycle MiSeq reagent kit version 2 (catalog number MS-102-2003; Illumina, Vancouver, Canada).

### Processing of sequence reads.

Sequence reads were demultiplexed using the MiSeq Reporter software version 2.5.0.5 (Illumina). Demultiplexed sequences were processed to generate amplicon sequence variants (ASVs) using QIIME2 (74), managed by Automation, eXtension, and Integration of Microbial Ecology (AXIOME) version 3.0 (75). Forward and reverse reads were trimmed to a shared nucleotide-515-to-805 V4 region across all data sets using primers 515F-Y and Pro805R (3′-GACTACNVGGGTATCTAATCC-5′) using cut-adapt (76) version 2019.10.0. Trimmed reads were denoised and merged and chimeras removed using DADA2 version 2019.10.0 (77) while maintaining a minimum of a 12-base overlap for the forward and reverse reads. The ASVs were classified using a naive Bayes classifier trained with the SILVA 132 SSURef NR99 database (78), with additional taxonomic annotation and reassignment using the GTDB in order to ensure compliance with SILVA 138 classifications (19). Additional 454 pyrosequencing data from a previous human skin microbiome study (“Roche 454”) (32) were imported as single-end reads through QIIME2 using the *qiime dada2 denoise-pyro* command and trimmed using a workflow identical to that used for the paired-end reads, excluding the merge-denoising step. Negative controls were manually inspected to ensure that they were distinct from sample profiles and were treated appropriately. Only two ASVs had overlaps among three controls and four nonhuman mammalian skin samples. Because these ASVs were observed in only a small number of controls (3/81) and had low read proportions for both the controls and samples, these ASVs were not removed. Because the Roche 454 data set was generated with archaeon-specific primers, these data are limited to analysis of the identities and distributions of archaeal genera rather than their relative abundances in relation to bacteria.

### Tree generation.

The 16S rRNA gene multiple sequence alignment of all archaeal ASVs was performed using ClustalW (79), with a gap opening penalty of 15.0 and a gap extension penalty of 6.66, in MEGA X version 10.1.8 (80). Sequence alignments were trimmed for gaps as appropriate. A maximum-likelihood tree was generated in MEGA X using a GTR +G +I nucleotide substitution model; confidence was assessed with 1,000 bootstraps.

### Assessment of primer coverage.

Universal prokaryotic and archaeon-specific primers used in the current and previous studies were analyzed *in silico* for their coverage of the domain *Archaea*. Forward and reverse primer sequences were entered into SILVA TestPrime version 1.0 using the SSU 138 database (81). Primers were tested without allowing mismatches (zero mismatch) and by allowing a single mismatch provided that it did not occur within five bases proximal to the 3′ end (one mismatch). Database coverage was parsed from the taxonomy browser into three categories: “*Bacteria*,” “*Archaea*,” and “*Nitrososphaeria*.”

### Data availability.

All 515F-Y/926R sequences generated for the current study were deposited in the European Nucleotide Archive (ENA) under project accession numbers PRJEB42587 (human/mammalian skin 92-sample subset) and PRJEB42589 (finger and computer keyboard swabs). Sequence data from projects with accession numbers PRJNA385010 (mammalian skin) (36), PRJNA345497 (human skin) (16), and PRJNA313528 (human skin) (32) were retrieved from the Sequence Read Archive. Sequence data from the project with accession number PRJEB10962 (campus door handles) (40) were retrieved from the European Nucleotide Archive.
